# Renoprotective effect of febuxostat on contrast-induced acute kidney injury in chronic kidney disease patients stage 3: randomized controlled trial

**DOI:** 10.1186/s12882-023-03114-4

**Published:** 2023-03-22

**Authors:** Iman Ibrahim Sarhan, Yasser A. Abdellatif, Rania Elsayed Saad, Nahla Mohamed Teama

**Affiliations:** 1 Department of Internal Medicine and Nephrology, Faculty of Medicine, Ain Shams University, Ramses Street 38, Abbasia, Cairo, 11566 Egypt; 2 Department of Cardiology, Faculty of Medicine, Ain Shams University, Cairo, Egypt; 3 Department of Nephrology, Elsahel teaching hospital, Cairo, Egypt

**Keywords:** Contrast induced acute kidney injury, Febuxostat, Percutaneous coronary intervention, Contrast

## Abstract

**Introduction:**

Contrast-induced acute kidney injury (CI-AKI) is known to be a complication of using intravascular contrast injection. Unfortunately, it is associated with adverse outcomes such as prolonged length of hospitalization and increased burden of health care costs. So, we aimed to determine the efficacy of febuxostat in the prevention of contrast-induced acute kidney injury among patients with chronic kidney disease Stage 3 performing percutaneous coronary intervention (PCI).

**Methods:**

In a randomized controlled trial we enrolled 120 CKD stage 3 Patients with acute coronary syndrome referred to the cardiology department Ain-Shams University hospital for performing PCI and stenting. Patients were randomly assigned to two arms: Group I (study group): Included 60 patients who received Febuxostat added to the traditional treatment (IV hydration and N-acetylcysteine). The patients received Feburic 80 mg within 6–18 h before and within 6–18 h after the coronary intervention (a time gap of 24 h between two doses). Group II (control group): included 60 patients who received only traditional treatment.

**Results:**

The incidence of AKI was higher in the control group with a statistically significant difference. We found that Independent Significant risk factors that led to AKI were febuxostate avoidance, DM, high urea level, high creatinine level, CKD stage 3B, high Mehran score and high AKI risk.

**Conclusion:**

We demonstrated that febuxostat has a Reno protective effect and it can help to reduce the incidence CI-AKI in CKD patients stage 3 performing PCI.

## Introduction

Contrast-induced acute kidney injury (CI-AKI) is considered the third commonest cause of acute kidney injury among hospitalized patients. The incidence of CI-AKI has gradually increased especially with percutaneous coronary intervention (PCI). It is more noticed among patients experiencing primary PCI due to hemodynamic instability and inadequate prophylaxis [1]. CI-AKI is often considered a transient event, as serum creatinine returns to its normal values within 1 to 3 weeks in 80% of patients. In some patients CI-AKI is associated with many adverse events, as progression to renal failure, and even the need for initiation of renal replacement therapy, prolongation of the length of hospital stay, cardiovascular events, and increased mortality rate [2]. There are three main mechanisms that lead to CI-AKI: Renal vasoconstriction and increased medullary hypoxia, formation of reactive oxygen species (ROS) and increased toxicity of the renal tubular cells. They ultimately lead to apoptosis of endothelial and epithelial cells in addition to reduction of glomerular filtration rate (GFR) [3]. Oxidative stress has a pivotal role in development of CI-AKI; contrast media has a detrimental effect on the antioxidant defense mechanism. The decline in GFR causes hypoperfusion of the renal medulla, and increased viscosity of the blood filtered by nephrons. Medullary hypoperfusion decreases the delivery of oxygen which in turn leads to ischemic injury of the renal tubules [4]. Accordingly, researchers have investigated several therapeutic interventions to target the mechanisms of this proposed hypothesis.

Uric acid is a biological factor which has been shown to be a neurostimulator, proinflammatory and activator of the innate immune response as well as have a pro- and antioxidant effect. These effects might provide a possible explanation for the association of uric acid with the development of chronic hypertension, diabetes mellitus, metabolic syndrome, coronary artery disease as well as chronic kidney disease. Regarding chronic kidney disease (CKD), several clinical studies have concluded that hyperuricemia is an important risk predictor for the onset and progression of CKD [5–11]. Uric acid-lowering therapy (ULT) targeting serum uric acid (SUA) successfully delays the decline of renal functions and its progression in CKD patients with hyperuricemia [12].

Recently, uric acid has been proposed to be a potential mediator of acute kidney injury (AKI) via both systemic effects of hyperuricemia and local effects via crystal-dependent mechanisms and crystal-independent mechanisms. In crystal-dependent mechanisms, hyperuricemia, especially in tumor lysis syndrome, leads to crystal-induced tubulopathy, with elevated tubular pressure and increased renal vascular resistance leading to a decline in GFR and eventually AKI. In the crystal-independent mechanisms, it has been experimentally shown that without crystal deposition, even mild hyperuricemia may increase the risk for AKI, via pro-inflammatory and anti-angiogenic mechanisms. Uric acid induces renin-angiotensin-aldosterone system (RAAS) activation, increased inflammatory mediators (MCP-1, ICAM), increased reactive oxygen radicals, decreased nitric oxide bioavailability, impaired renal autoregulation, and subsequent decline in GFR. All these postulated effects can potentially increase the susceptibility for AKI [13–15]. Moreover, the fundamental changes in vasoconstrictive mechanisms and renal vasculature that occur in AKI are similar to those observed with hyperuricemia. According to this hypothesis, researchers have investigated whether uric acid-lowering agents could provide renoprotection by reducing the risk of AKI.

Febuxostat, a non-purine selective xanthine oxidase inhibitor, has a strong hypouricemic effect with no serious negative outcomes, even for patients with CKD stages 1–3. Febuxostat can also have reno-protective effects in addition to the urate-lowering therapy due to its inhibitory effect on oxidative stress and inflammatory biomarkers [16].

Some clinical studies were conducted to compare the reno-protective and uric acid-lowering therapy effects between febuxostat and allopurinol among CKD patients [17–19]. Zhuang, Lihua compared the reno-protective effects of both Febuxostat and allopurinol among patients with combined chronic kidney disease and hyperuricemia. They reported that febuxostat was better than allopurinol in delaying the deterioration of renal function among those patients with both CKD and hyperuricemia [20]. In our study, we aimed to investigate the reno-protective effect of febuxostat in the prevention of contrast-induced acute kidney injury in stage 3 CKD patients undergoing percutaneous coronary intervention (PCI).

## Methods

In a randomized controlled trial, we enrolled 120 CKD stage 3 Patients with acute coronary syndrome referred to the cardiology department Ain-Shams University hospital for performing PCI and stenting starting from 25 to 2022 to the second of September 2022. This study was performed in accordance with the ethical standards of Ain Shams University Research Committee and with the 1964 Helsinki declaration and its later amendments or comparable ethical standards. Ain Shams University Faculty of Medicine Research Ethics Committee (REC) FWA 000017585 has approved the study protocol with approval number (MS 616/2020). The clinical trial registration number is NCT05264584, and the first date of registration was 03/03/2022. An informed consent was obtained from the included patients. All included patients met the inclusion criteria; were older than 18 years old, CKD patients’ stage 3, under treatment of high dose statin; Atorvastatin (40–80 mg/day) and undergoing PCI. Patients with known allergy to febuxostat or under treatment with uric acid-lowering medications (allopurinol- febuxostat-benzbromarone) and patients with severe debilitating diseases like liver cirrhosis, hypoalbuminemia or heart failure were excluded. Patients who developed hemodynamic instability periprocedurally, also were excluded from our study. In addition, those patients who underwent high osmolar contrast media during the procedure were also excluded. All patients received low osmolar contrast media and IV hydration in the form of a continuous intravenous infusion of isotonic saline at a rate of 0.5 mL/kg/h for at least 2 to 12 h before the procedure and lasted for 6 to 24 h afterward. They also received N-acetylcysteine 600 mg orally twice daily on the day before and the day of exposure to contrast or a single periprocedural dose (1200 mg, within 4 h of contrast exposure). Patients were randomly assigned to two arms.

### Study group

included 60 patients who received Febuxostat 80 mg within 6–18 h before the procedure and 6–18 h after the coronary intervention with a time gap of 24 h between the two doses. This treatment will be added to the traditional treatment (IV hydration & N-acetylcysteine).

### Control group

included 60 patients who received traditional treatment only.

All patients were subjected to comprehensive history taking with particular emphasis on age, sex, co-morbidities, and drug history. Laboratory investigations were performed to all patients, including:

- Preoperative: CBC, blood urea, creatinine, uric acid, lipid profile, ALT, AST, Sodium, potassium, calcium, phosphorus and urine analysis.
- 3 to 5 days post-operative: Serum creatinine, blood urea, and uric acid.

The Mehran risk score was calculated, and the predicted risk of AKI was subsequently calculated. The Mehran risk score is a scoring system based on awarded to each of the following multivariate predictors: (hypotension = 5 points; intra-aortic balloon pump (IABP) use = 5 points; congestive heart failure = 5 points; serum creatinine > 1.5 mg/dL = 4 points.

age > 75 years = 4 points; anemia = 3 points; diabetes mellitus = 3 points; contrast volume = 1 point for each 100 mL used) [21].

The predicted AKI-risk was calculated based on the calculated total Mehran score according to the risk categories by Mehran et al.:

Low risk (score of ≤ 5): CIN rate 7.5%, dialysis in 0.04%.

Moderate risk (score of 6–10): CIN rate 14%, dialysis in 0.12%.

High risk (score of 11–15): CIN rate 26.1%, dialysis in 1.09%,

Very high risk (score of ≥ 16): CIN rate 57.3%, dialysis in 12.6% [21].

The primary endpoint of the study was the incidence of CI-AKI which was defined as an increase in serum creatinine by an increase in serum creatinine of ≥ 0.3 mg/dl, or a serum creatinine increase of ≥ 1.5– 1.9 times baseline occurs within 3 days following the intravascular administration of a contrast medium in the absence of an alternative etiology.

### Sample size calculation

After reviewing the literature [22], we used G power 3.0.10 (Franz Faul, University at Kiel, Germany) for windows for sample size calculation. With a power of 80%, α = 0.05, an effect size of 0.5 and allocation ratio of 1:1. We found that the minimum required sample size per group would be 64 patients per group. However, 4 patients were lost from follow up in each group.

So, 2 groups of patients were allocated; Group 1 included 60 patients with CKD stage 3 undergoing PCI using Febuxostat in addition to traditional treatment and Group 2 with same underlying conditions and received traditional treatment only 24 h before the procedure.

Block randomization was used in this clinical trial design to reduce bias and achieve balance in the allocation of participants to treatment arms and it was open label.

## Statistical analysis

We used SPSS (statistical package for social sciences) version 24 for analyzing the data. Numerical data was described in terms of mean and standard deviations if normally distributed and median and interquartile ranges if non normally distributed. Qualitative data was described in terms of frequencies and percentages. Chi square test was used to test the association between categorical variables (Chi- square yields an unadjusted odds ratio with 95% confidence interval). Fisher exact test was used in case of violation of the assumptions. Independent sample t test was used to test the difference between two groups concerning normally distributed numerical variables (t statistics were used to calculate confidence interval). To test the effect of independent variables on dependent variables, binary logistic regression was performed (Odds ratio was calculated for data of categorical type and exponential beta for data of continuous type). P-value less than 0.05 was considered statistically significant.

## Results

**Fig. 1:**
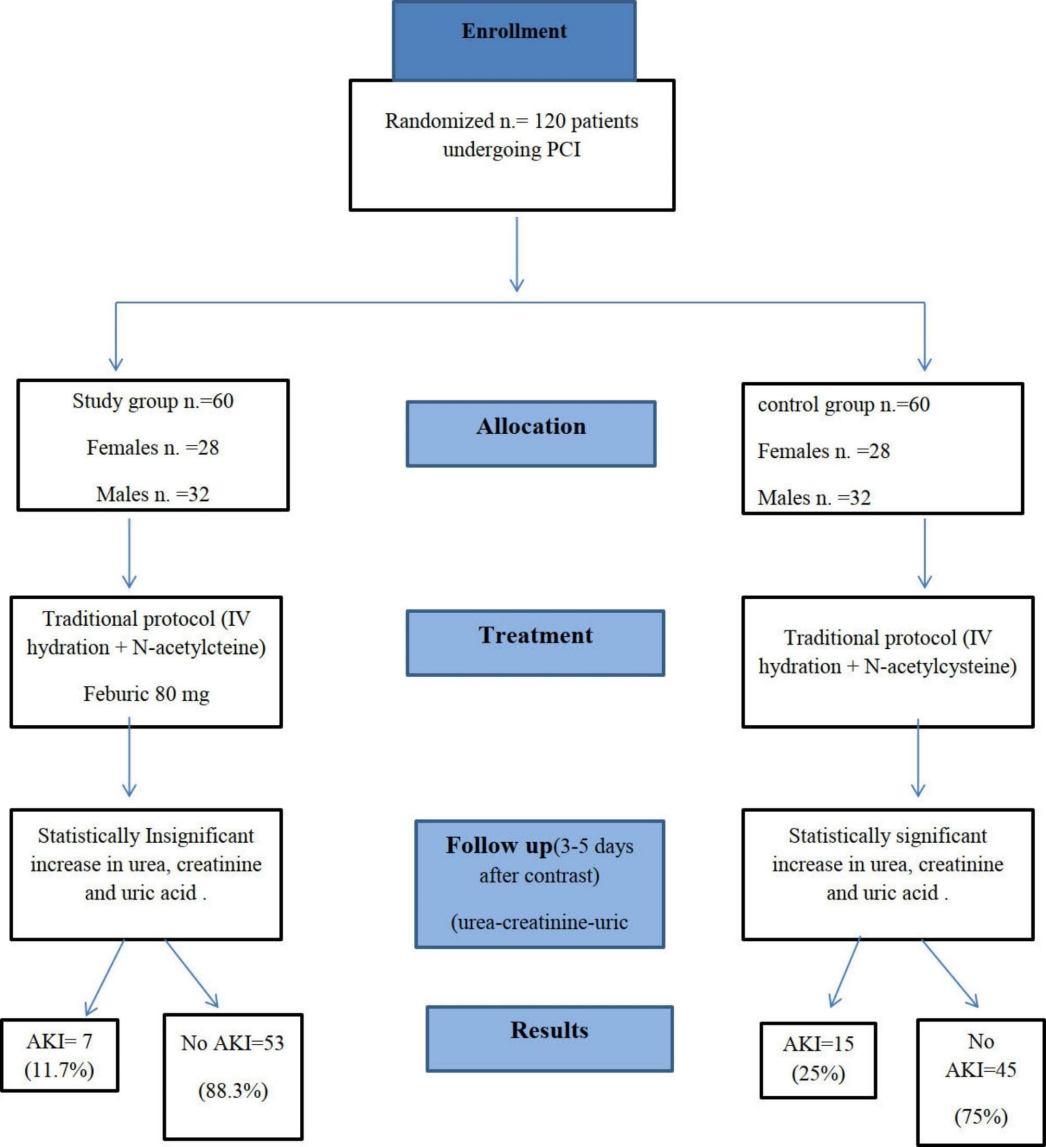
CONSORT flow diagram

We enrolled 120 CKD stage 3 patients with acute coronary syndrome referred to the cardiology department, Ain shams university hospital for performing PCI. We used block randomization technique to randomize subjects into groups that result in equal sample sizes. Patients were randomized into two arms (control group & study group). Each arm consisted of 60 patients. The control group received IV hydration and N-acetylcysteine. The study group received IV hydration, N-acetylcysteine and febuxostat (Fig. 1). The patients were followed up for 3–5 days for occurrence of CI-AKI. The consort flow diagram of the study is shown in Fig. (1).

The baseline demographics and associated co-morbidities were well balanced between both groups. There were no significant differences between both groups regarding the demographics and associated comorbidities. There were no significant differences between both groups regarding baseline laboratory results. In addition, there were no significant differences regarding baseline ACEI, ARBS or SGLT2 inhibitors administration. (Table 1)

**Table 1:** Comparison between cases and controls concerning demographics and associated comorbidities, baseline laboratory investigations, and baseline relevant medications

Variable	Study group (60 patients) Cases (n = 60)	Control group (60 patients)	95% confidence Interval		Test of Significance P value
			Lower	Upper	
**Demographics and co-morbidities**					
Age (years)	61.07 ± 7.3	58.9 ± 7.2	-4.7	0.45	t= -1.64 p = 0.105
Gender, No. (%) Male Female	32 (53.3%) 28 (46.7%)	32 (53.3%) 28 (46.7%)	0.78	1.642	X^2^ = 0.8 p = 1.00
BMI (Kg/m^2^)	31.5 ± 3.8	31.1 ± 3.4	-1.5	0.98	t= -0.46 p = 0.506
DM, No. (%)	22 (36.7%)	25 (41.7%)	-0.5	0.87	X^2^ = 0.57 p = 0.709
HTN, No. (%)	37 (61.7%)	36 (60%)	-0.6	2.2	X^2^ = 0.035 p = 0.8
Dyslipidemia, No. (%)	38 (63.3%)	38 (63.3%)	1.2	2.3	X^2^ = 0.035 p = 0.8
Stroke, No. (%)	7 (11.7%)	6 (10%)	-0.11	0.27	F = 0.035 p = 0.8
IBD, No. (%)	0 (0%)	1 (1.7%)	-0.49	0.09	F = 0.035 p = 0.8
Malignancy, No. (%)	1 (1.7%)	2 (3.3%)	0.008	0.09	F = 0.035 p = 0.8
**Baseline laboratory investigations**					
Urinalysis No. (%) - Proteinuria - Hematuria - Pus cells	33 (55%) 14 (23.33%) 16 (26.67%)	29 (48.33%) 17 (28.33%) 21 (35%)	0.77 0.2 0.25	1.64 0.7 0.67	X^2^ = 0.35; p = 0.46 X^2^ = 0.3; p = 0.56 X^2^ = 0.45; p = 0.76
Protein/ creatinine ratio	2.7 ± 1.2	2.4 ± 1.15	-0.73	0.12	t= -1.39 p = 0.16
Hb (g/dl)	11.04 ± 0.8	11.3 ± 1.08	-0.083	0.6	t = 1.48 p = 0.13
Platelets	239.8 ± 63.97	238.5 ± 52.28	-21.8	19.88	t= -0.095 p = 0.33
WBCs	7.33 ± 1.81	7.27 ± 1.81	-0.7	0.59	t= -0.18 p = 0.848
Serum urea (mg/dl)	49.58 ± 11.5	48.6 ± 12.6	-5.34	3.38	t= -0.45 p = 0.656
Serum creatinine (mg/dl)	1.59 ± 0.19	1.64 ± 0.27	-0.034	0.13	t = 1.17 p = 0.276
Serum uric acid (mg/dl)	5.74 ± 1.39	5.46 ± 1.18	-0.74	0.18	t= -1.19 p = 0.230
Total Cholesterol (mg/dl)	195.32 ± 16.32	193.7 ± 18.6	-7.94	4.7	t= -0.51 p = 0.61
Triglycerides (mg/dl)	146.12 ± 15.9	145.56 ± 18.7	-6.89	5.65	t= -0.196 p = 0.9
HDL (mg/dl)	60.03 ± 8.3	59.18 ± 9.32	-4.04	2.34	t= -0.52 p = 0.598
LDL (mg/dl)	124.2 ± 13.7	124.7 ± 16.5	-4.98	5.98	t = 0.18 p = 0.862
ALT (U/L)	30.27 ± 6.9	30.35 ± 9.5	-2.9	3.08	t = 0.053 p = 0.1
AST (U/L)	40.28 ± 11.5	39.85 ± 10.8	-4.4	3.7	t= -0.21 p = 0.2
Serum sodium (mg/dl)	140.3 ± 3.7	140.17 ± 2.7	-1.3	1.04	t= -0.22 p = 0.823
Serum potassium (mg/dl)	4.6 ± 0.6	4.5 ± 0.61	-0.32	0.12	t= -0.91 p = 0.351
Total calcium(mg/dl)	8.91 ± 0.77	9.01 ± 0.61	-0.14	0.36	t = 0.86 p = 0.440
Serum Phosphorous (mg/dl)	3.53 ± 0.85	3.62 ± 0.89	-0.22	0.4	t = 0.56 p = 0.15
Estimated GFR (ml/min./1.73 m^2^)	47.7 ± 7.75	46.88 ± 7.14	-3.5	1.87	t= -0.6 p = 0.543
CKD stage, No. (%) Stage 3 A Stage 3B	41 (68.3) 19 (31.7)	34 (56.7) 26 (43.3)	1.54	2.9	X^2^ = 0.35 p = 0.258
**Baseline relevant medications**					
ACEI	26 (43.3%)	23 (38.3%)	0.49	1.12	X^2^ = 0.3 p = 0.57
ARBs	19 (31.6%)	22 (36.67%)	0.28	0.7	X^2^ = 0.3 p = 0.56
SGLT2 inhibitors	18 (30%)	20 (33.3%)	0.25	0.67	X^2^ = 0.15 p = 0.69

X^2^; chi-square test, F; Fisher exact test, T; Independent sample T-test.

In the study group, there were no statistically significant difference between pre- and post- contrast exposure as regards serum, urea, creatinine and uric acid (Table 2). However, in the control group, although there was no statistical difference in serum uric acid between pre- and post- contrast exposure, there were statistically significant difference in serum urea (p = 0.0001) and serum creatinine (p = 0.014) as shown in Table 3.

**Table 2:** Comparison between both groups as regards renal function tests after contrast

Variable	Pre-contrast	Post-contrast	95% confidence interval		P value
			Lower	Upper	
Urea (mg/dL)	55.2 ± 17.35	62.93 ± 12.5	3.8	11.6	t = 3.9 ***p = 0.006***
Creatinine (mg/dL)	1.75 ± 0.77	2.1 ± 0.96	0.12	0.57	t = 3.12 ***p = 0.02***
Uric acid (mg/dL)	5.83 ± 1.86	5.57 ± 2.42	-0.81	0.29	t= -0.93 p = 0.5

**Table 3:** Comparison of renal function parameters in the study group between before and after contrast

Variable	Pre-contrast	Post-contrast	95% confidence interval		P value
			Lower	Upper	
Urea (mg/dL)	49.58 ± 11.47	55.2 ± 17.35	0.3	10.9	t = 2.09 ***p = 0.04***
Creatinine (mg/dL)	1.59 ± 0.19	1.75 ± 0.77	-0.04	0.36	t = 1.56 p = 0.23
Uric acid	5.74 ± 1.4	5.83 ± 1.86	-0.5	0.68	t = 0.299 p = 0.76

It is worth mentioning that there was statistically significant difference between both groups regarding post-contrast urea (p-value: 0.006) and creatinine (p-value: 0.02) as urea and creatinine were higher among control group. While there was no statistically significant difference as regard uric acid (p value:0.5) as shown in (Table 4).

**Table 4:** Comparison of renal function parameters in the control group between before and after contrast

Variable	Pre-contrast	Post-contrast	95% confidence interval		P value
			Lower	Upper	
Urea (mg/dL)	48.6 ± 12.6	62.93 ± 12.5	9.79	18.86	t = 6.25 ***p = 0.0001***
Creatinine (mg/dL)	1.64 ± 0.27	2.1 ± 0.96	0.2	0.7	t = 3.57 ***p = 0.014***
Uric acid (mg/dL)	5.46 ± 1.2	5.57 ± 2.42	-0.58	0.8	t = 32 p = 0.824

As regard the degree of change in creatinine and urea before and after contrast, there was statistically significant difference between both groups (p value: 0.0001) but there was no significant change between both groups as regard uric acid (Table 5).

**Table 5:** Comparison between degree of changes in renal function parameters in both groups

Variable	Study group (60 patients) Cases (n = 60)	Control group (60 patients)	95% confidence interval		P value
			Lower	Upper	
Urea (mg/dL)	11.2 ± 4.6	14.6 ± 3.2	1.96	4.84	t = 4.7 ***p = 0.0001***
Creatinine (mg/dL)	0.15 ± 0.02	0.3 ± 0.06	0.13	0.17	t = 18.37 ***p = 0.0001***
Uric acid (mg/dL)	0.1 ± 0.04	0.13 ± 0.04	0.015	0.045	t = 4.1 ***p = 0.3***

There was no statistically significant difference between both groups regarding the calculated Mehran score and the predicted CI-AKI-risk. However, the incidence of AKI was significantly lower in the study group (p value: 0.048), as shown in (Table 6).

**Table 6:** The difference between Study and control groups concerning CKD stage and volume of contrast administrated for all patients

Variable	Study group	Control group	95% confidence interval		P value
			Lower	Upper	
Contrast volume, ml	185.83 ± 41.6	190.17 ± 42.6	-10.8	19.6	t = 0.57 p = 0.128
Mehran score	5.7 ± 2.7	5.52 ± 2.5	-1.12	0.76	t= -0.38 p = 0.582
Predicted CI-AKI risk (%)	12.4 ± 6.94	10.87 ± 4.23	-3.6	0.54	t= -1.45 p = 0.139
CI-AKI Development, No. (%)	7 (11.7%)	15 (25%)	0.13	0.4	X^2^ = 4.2 ***p = 0.048***

X^2^; Chi square test. T; independent sample t test.

According to the incident CI-AKI, the whole study population (120 patients) was divided into two groups, the CI-AKI group (22 patients) and the non-CI-AKI group (98 patients). We subsequently compared both groups regarding demographics, co-morbidities, baseline laboratory investigations, baseline relevant medications including Febuxostat use, contrast volume, Mehran score and predicted CI-AKI-risk (Table 7).

**Table 7:** Univariate analysis of factors affecting CI-AKI

	CI-AKI group (22 patients)	Non- CI-AKI group (98 patients)	95% confidence interval			P value
			Lower		Upper	
***Demographics and co-morbidities***						
**Age (years)** mean ± SD	61.54 ± 6.4	59.65 ± 7.5	-5.3	1.5		t= -1.095 p = 0.299
**Gender, number (%)** Male Female	12 (54.5%) 10 (45.5%)	52 (53.1%) 46 (46.9%)	0.3	0.9		X^2^ = 0.2 p = 0.9
**BMI (Kg/m**^**2**^**)**, mean ± SD	30.83 ± 4.7	31.4 ± 3.3	-1.08	2.3		t = 0.71 p = 0.51
DM	14 (63.6%)	33 (33.7%)	0.23	1.067		X^2^ = 6.7 ***p = 0.009***
HTN	17 (77.3%)	56 (57.1%)	0.4	1.2		X^2^ = 3.05 p = 0.08
Dyslipidemia	16 (72.7%)	60 (61.2%)	0.46	1.18		X^2^ = 1.02 p = 0.3
Stroke	2 (9.1%)	11 (11.2%)	0.056	0.3		F = 0.08 p = 0.7
Malignancy	0 (0%)	3 (3.1%)	0.006	0.17		X^2^ = 0.02 p = 0.4
***Baseline laboratory investigations***						
Urea	54.59 ± 9.1	47.85 ± 12.27	***-12.2***	***-1.2***		t= -2.4 ***p = 0.017***
Creatinine	1.8 ± 0.26	1.5 ± 0.19	***-0.39***	***-0.2***		t= -6.22 ***p = 0.0001***
Uric acid	5.7 ± 1.15	5.56 ± 1.3	-0.74	0.455		t= -0.466 p = 0.471
Cholesterol	194.22 ± 16.93	194.59 ± 17.66	-7.86	8.46		t = 0.073 p = 0.930
Triglycerides	141.22 ± 25.04	143.39 ± 15.6	-6.07	10.42		t = 0.53 p = 0.604
HDL	59.22 ± 9.4	59.69 ± 8.69	-3.65	4.5		t = 0.23 p = 0.823
LDL	122.22 ± 15.7	124.93 ± 15.01	-4.3	9.77		t = 0.76 p = 0.449
eGFR	42.29 ± 6.6	48.4 ± 7.1	***2.8***	***9.38***		t = 3.69 ***p = 0.0001***
**CKD stage No. (%)** 3 A 3B	8 (36.4%) 14 (63.6%)	67 (68.4%) 31 (31.6%)	0.53	3.7		X^2^ = 7.8 ***p = 0.005***
***Relevant baseline medications***						
ACEI	11 (50%)	38 (38.77)	0.27	0.89		X^2^ = 0.9 p = 0.45
ARBs	7 (31.8%)	34 (34.7%)	0.24	0.65		X^2^ = 0.06 p = 0.83
SGLT2 inhibitors	6 (27.27%)	32 (32.6%)	0.22	0.67		X^2^ = 0.17 p = 0.8
Febuxostat use	7 (31.8%)	53 (54.1%)	0.1	0.7		X^2^ = 4.2 ***p = 0.048***
***Contrast volume, Mehran score and predicted CI-AKI-risk***						
Contrast volume, mean ± SD	184.09 ± 52.7	180.9 ± 41.27	-23.5	17.14		t= -0.31 p = 0.758
Mehran score, mean ± SD	8.13 ± 3.6	5.09 ± 2	***-4.14***	***1.9***		***t= -8.2*** ***p = 0.001***
Predicted CI-AKI-risk (%) mean ± SD	16.4 ± 3.3	10.57 ± 3.23	***-7.34***	***-4.3***		***t= -7.6*** ***p = 0.001***

There were no statistically significant differences between both groups regarding demographics and comorbidities except for diabetes mellitus which was significantly more frequent in the CI-AKI group (p-value: 0.009).

Regarding the renal function parameters, preprocedural serum creatinine and urea were significantly higher among the CI-AKI group (p-value: 0.0001; 0.017, respectively). while eGFR was significantly lower among the CI-AKI group (p value: 0.0001). In addition, a significantly higher proportion of the CI-AKI group was in stage 3B at baseline compared to the non-CI-AKI group with a higher proportion in stage 3 A (p = 0.005). However, both groups had no statistically significant difference in serum uric acid.

Regarding relevant preprocedural medications, although there were no statistical differences between both groups as regards ACEI, ARBs or SGLT2 inhibitors, the proportion of patients who received Febuxostat peri procedurally was significantly higher among the non-CI-AKI group (p-value: 0.048). As regards procedural contrast, both groups were comparable (p = 0.758).

The Mehran score and predicted CI-AKI-risk were significantly higher in the CI-AKI group (both with p-value 0.001) (Table 7).

From the previous analysis, eight risk factors for AKI were reported. These risk factors then were analyzed using multi-variate regression analysis. Three factors were able to keep their significance as independent predictors for occurrence of CI-AKI: febuxostat avoidance (p-value: 0.048), baseline serum creatinine (p-value: 0.0001) and Mehran score (p-value: 0.027) (Table 8).

**Table 8:** Multiple logistic regression analysis

	ß estimate	95% CI (lower)	95% CI (upper)	OR / Exponential Beta	P value
**Diabetes mellitus**	0.006	-0.129	140	3.4	0.932
**Serum Urea**	-0.003	-0.009	0.003	0.107	0.258
**Serum Creatinine**	0.668	0.336	1.00	0.402	***0.0001***
**Estimated GFR**	-0.011	-0.025	0.004	0.203	0.153
**CKD-stage**	-0.091	-0.313	0.132	0.26	0.422
**Mehran score**	0.5	0.006	0.095	0.342	***0.027***
**AKI-risk**	0.006	0.089	-0.013	0.025	0.533
**Febuxostat use**	-0.123	-0.240	-0.006	0.39	***0.048***

CI = confidence interval; OR = Odds Ratio.

## Discussion

Besides being relatively inexpensive, our study showed that febuxostat is safe and tolerable in this patient subset. No adverse reactions occurred in the febuxostat group, including cardiovascular events and severe allergic reactions.

Febuxostat is a non-purine analog xanthine oxide reductase (XOR) inhibitor that showed better organ protection than allopurinol. The superior efficacy of non-purine analog inhibitors was due to their greater inhibition of XOR activity, which reduced oxidative stress. Febuxostat has also been suggested to inhibit inflammation and apoptosis through MAPK signaling [23].

In our study, the mean age was 60 ± 7.32 years old. However, age didn’t affect the incidence of CI-AKI this could be due to age matching between the study and control group, which is similar to the findings of a study by Ma, Li [24]. Contrary to our study, Zhuang, Lihua and Pavasini et al., reported age as a significant risk factor for CI-AKI [20, 25]. In the current study, there was no significant influence of either gender or BMI on the incidence of CI-AKI which come in agreement with the results of Lee and Lee [19].

The Febuxostat dose was 80 mg 6–18 h before and after the procedure with a time gap of 24 h between the two doses. Different doses were reported in other studies. Kamet et al. showed that the hypouricaemic effect of febuxostat was evident within an hour of drug administration. The nadir of serum urate was highly variable but was achieved at an average of 24 ± 16 h after dosage [26]. Different doses and frequencies were used in other studies. Ma, Li used 40 mg P.O. 1 day before the procedure and three days after the procedure [24]. Zhuang, Lihua used 40 mg P.O. one day before and one day after the procedure [20]. In the current study, both groups were comparable regarding baseline characteristics, associated medical disorders and laboratory investigations including uric acid, serum creatinine, eGFR and CKD stages also they were comparable.

In the current study, we used serum creatinine and estimated GFR as biomarkers for renal impairment. Estimated GFR was measured using CKD-EPI equation. The total contrast volume needed in our study was 187.25 ± 42.36 ml and both groups were comparable between both groups. This is similar to the contrast volume used for PCI in other [20, 24, 27]. The Contrast dose did not affect the incidence of CI-AKI in our study, which is similar to the findings of Ma, Li. However, contrast dose was reported as a risk factor in Zhuang, Lihua [20].This could be explained as all our patients received comparable doses of contrast.

Diabetes was reported to be a risk factor for CI-AKI development in the current study. However, in multivariate analysis, diabetes lost its significance when adjusted to the other risk factors. Adjusted risk factors in a study by Ma, Li did not also report diabetes as significant factor [24]. Many researchers recommended usage of a scoring system for assessment of CI-AKI risk to identify the high-risk groups, and improve the outcomes [28]. In our current study, two scores were used; Mehran score and AKI-risk scores and the validity in predicting incidence of CI-AKI was assured with presence of statistically significant difference between AKI and no AKI groups regarding both scores.

In the current study, there was a statistically significant difference between both groups regarding the incidence of CI-AKI as AKI occurred in 18.3%, including 25% in the control group and only 11.7% in the study group (Febuxostat group) (p-value: 0.048). However, Ma et al. reported that CI-AKI occurred in 10.4%, including 6% in the febuxostat group and 14.71% in the control group [24]. The difference in the AKI incidence in the different studies may be attributed to the different study populations as all the included patients in our study were CKD stage 3, a strong predictor of CI-AKI, as well as different doses and frequency of Febuxostat used. CI-AKI incidence was more frequent among the CKD stage 3B group and less frequent in the 3 A this was in accordance with Latcha et al. who also reported that congestive heart failure and previous AKI can also increase the risk for CI-AKI [29].

There was no statistically significant difference between both groups regarding serum uric acid before and after the procedure. Contrary to our study, serum uric acid decreased significantly in the Febuxostat group in both previous studies that were performed by Ma, Li and Zhuang, Lihua [20, 24]. This difference is attributed to the timing of uric acid postprocedural testing, being 3–5 days in our study in contrast to 48 h in Ma et al. study and 72 h in Zhuang et al. study. It is possible that serum urate concentrations recovered gradually to near the baseline concentrations over about three days, as shown by Kamel, Graham [26].

Multivariate logistic regression analysis was conducted to identify factors related to development of CI-AKI. Only baseline serum creatinine, Mehran score, and febuxostat avoidance kept their significance as independent predictors of CI-AKI incidence in the multivariate model. This result is concordant with the study of Ma, Li [24] but discordant with the result of Zhuang, Lihua [20].

As regard the use of drugs which may affect the incidence of CI AKI as SGL2I, ACEI or ARBs this come in consistency with the findings of Pavasini et al., who demonstrated that using these medications as a baseline medications is not considered as an independent predictors of CA-AKI [25].

The study had some limitations. It is a single-center study that was confined to CKD stage 3 patients and lacked follow-up for mortality, need for dialysis or prolonged hospitalization. The study had the advantage of being prospective, randomized. Using block randomization decreases the incidence of selection bias. Sample size was adequate in comparison to the recent studies evaluating the same topic. Different risk factors were studied and adjusted to the use of Febuxostat.

## Conclusion

We demonstrated that Febuxostat has a reno-protective effect. It can reduce CI-AKI incidence among CKD stage 3 patients undergoing PCI. Despite the limited studies regarding the use of Febuxostat in protection against CI-AKI among CKD patients, we recommend its use in CKD stage 3 patients, especially patients with hyperuricemia. We recommend using risk scores for acute kidney injury before giving contrast to CKD patients to stratify the patients and individualize contrast type, volume and preventive measures according to the results of these scores.

## Data Availability

The datasets used and/ or analyzed during the current study are available from the corresponding author on reasonable request.

## References

[1] Sun G, Chen P, Wang K, Li H, Chen S, Liu J et al. Contrast-Induced Nephropathy and Long-Term Mortality After Percutaneous Coronary Intervention in Patients With Acute Myocardial Infarction. Angiology [Internet]. 2019 Aug 1 [cited 2023 Feb 14];70(7):621–6. Available from: https://pubmed.ncbi.nlm.nih.gov/30317864/10.1177/0003319718803677 30317864

[2] Azzalini L, Kalra S. Contrast-Induced Acute Kidney Injury-Definitions, Epidemiology, and Implications. Interv Cardiol Clin [Internet]. 2020 Jul 1 [cited 2023 Feb 14];9(3):299–309. Available from: https://pubmed.ncbi.nlm.nih.gov/32471671/10.1016/j.iccl.2020.02.001 32471671

[3] Vlachopanos G, Schizas D, Hasemaki N, Georgalis A. Pathophysiology of Contrast-Induced Acute Kidney Injury (CIAKI). Curr Pharm Des [Internet]. 2019 Dec 11 [cited 2023 Feb 14];25(44):4642–7. Available from: https://pubmed.ncbi.nlm.nih.gov/31820694/10.2174/1381612825666191210152944 31820694

[4] Morcos R, Kucharik M, Bansal P, Al Taii H, Manam R, Casale J et al. Contrast-Induced Acute Kidney Injury: Review and Practical Update.Clin Med Insights Cardiol [Internet]. 2019 [cited 2023 Feb 14];13. Available from: /pmc/articles/PMC6826945/10.1177/1179546819878680 PMC6826945 31700251

[5] Von Lueder TG, Girerd N, Atar D, Agewall S, Lamiral Z, Kanbay M et al. Serum uric acid is associated with mortality and heart failure hospitalizations in patients with complicated myocardial infarction: findings from the High-Risk Myocardial Infarction Database Initiative. Eur J Heart Fail [Internet]. 2015 Nov 1 [cited 2023 Feb 14];17(11):1144–51. Available from: https://pubmed.ncbi.nlm.nih.gov/26424212/10.1002/ejhf.419 26424212

[6] Kanbay M, Snchez-Lozada LG, Franco M, Madero M, Solak Y, Rodriguez-Iturbe B et al. Microvascular disease and its role in the brain and cardiovascular system: a potential role for uric acid as a cardiorenal toxin. Nephrol Dial Transplant [Internet]. 2011 Feb 1 [cited 2023 Feb 14];26(2):430–7. Available from: https://academic.oup.com/ndt/article/26/2/430/1894767 10.1093/ndt/gfq635 20935013

[7] Kanbay M, Afsar B, Siriopol D, Unal HU, Karaman M, Saglam M et al. Relevance of uric acid and asymmetric dimethylarginine for modeling cardiovascular risk prediction in chronic kidney disease patients. Int Urol Nephrol [Internet]. 2016 Jul 1 [cited 2023 Feb 14];48(7):1129–36. Available from: https://pubmed.ncbi.nlm.nih.gov/27007614/10.1007/s11255-016-1271-6 27007614

[8] Kanbay M, Solak Y, Dogan E, Lanaspa MA, Covic A. Uric acid in hypertension and renal disease: the chicken or the egg? Blood Purif [Internet]. 2010 Dec [cited 2023 Feb 14];30(4):288–95. Available from: https://pubmed.ncbi.nlm.nih.gov/21088389/10.1159/000321074 21088389

[9] Kanbay M, Segal M, Afsar B, Kang DH, Rodriguez-Iturbe B, Johnson RJ. The role of uric acid in the pathogenesis of human cardiovascular disease. Heart [Internet]. 2013 Jun [cited 2023 Feb 14];99(11):759–66. Available from: https://pubmed.ncbi.nlm.nih.gov/23343689/10.1136/heartjnl-2012-302535 23343689

[10] Johnson RJ, Nakagawa T, Jalal D, Sánchez-Lozada LG, Kang DH, Ritz E. Uric acid and chronic kidney disease: which is chasing which? Nephrol Dial Transplant [Internet]. 2013 Sep [cited 2023 Feb 14];28(9):2221–8. Available from: https://pubmed.ncbi.nlm.nih.gov/23543594/10.1093/ndt/gft029 PMC4318947 23543594

[11] Nakagawa T, Kang DH, Feig D, Sanchez-Lozada LG, Srinivas TR, Sautin Y et al. Unearthing uric acid: an ancient factor with recently found significance in renal and cardiovascular disease. Kidney Int [Internet]. 2006 May [cited 2023 Feb 14];69(10):1722–5. Available from: https://pubmed.ncbi.nlm.nih.gov/16598194/10.1038/sj.ki.5000391 16598194

[12] Hahn K, Kanbay M, Lanaspa MA, Johnson RJ, Ejaz AA. Serum uric acid and acute kidney injury: A mini review. J Adv Res [Internet]. 2017 Sep 1 [cited 2023 Feb 14];8(5):529–36. Available from: https://pubmed.ncbi.nlm.nih.gov/28748118/10.1016/j.jare.2016.09.006 PMC5512150 28748118

[13] Talaat KM, El-Sheikh AR. The effect of mild hyperuricemia on urinary transforming growth factor beta and the progression of chronic kidney disease. Am J Nephrol [Internet]. 2007 Sep [cited 2023 Feb 14];27(5):435–40. Available from: https://pubmed.ncbi.nlm.nih.gov/17622758/10.1159/000105142 17622758

[14] Joung KW, Jo JY, Kim WJ, Choi DK, Chin JH, Lee EH et al. Association of preoperative uric acid and acute kidney injury following cardiovascular surgery. J Cardiothorac Vasc Anesth [Internet]. 2014 Dec 1 [cited 2023 Feb 14];28(6):1440–7. Available from: https://pubmed.ncbi.nlm.nih.gov/25245579/10.1053/j.jvca.2014.04.020 25245579

[15] Shimada M, Dass B, Ejaz AA. Paradigm shift in the role of uric acid in acute kidney injury. Semin Nephrol [Internet]. 2011 Sep [cited 2023 Feb 14];31(5):453–8. Available from: https://pubmed.ncbi.nlm.nih.gov/22000653/10.1016/j.semnephrol.2011.08.010 22000653

[16] Hira D, Chisaki Y, Noda S, Araki H, Uzu T, Maegawa H et al. Population Pharmacokinetics and Therapeutic Efficacy of Febuxostat in Patients with Severe Renal Impairment. Pharmacology [Internet]. 2015 Aug 20 [cited 2023 Feb 14];96(1–2):90–8. Available from: https://pubmed.ncbi.nlm.nih.gov/26183164/10.1159/000434633 26183164

[17] Chou HW, Chiu HT, Tsai CW, Ting IW, Yeh HC, Huang HC et al. Comparative effectiveness of allopurinol, febuxostat and benzbromarone on renal function in chronic kidney disease patients with hyperuricemia: a 13-year inception cohort study. Nephrol Dial Transplant [Internet]. 2018 [cited 2023 Feb 14];33(9):1620–7. Available from: https://pubmed.ncbi.nlm.nih.gov/29165620/10.1093/ndt/gfx313 29165620

[18] Liu X, Wang H, Ma R, Shao L, Zhang W, Jiang W et al. The urate-lowering efficacy and safety of febuxostat versus allopurinol in Chinese patients with asymptomatic hyperuricemia and with chronic kidney disease stages 3–5. Clin Exp Nephrol [Internet]. 2019 Mar 15 [cited 2023 Feb 14];23(3):362–70. Available from: https://pubmed.ncbi.nlm.nih.gov/30291473/10.1007/s10157-018-1652-5 30291473

[19] Lee JW, Lee KH. Comparison of renoprotective effects of febuxostat and allopurinol in hyperuricemic patients with chronic kidney disease. Int Urol Nephrol [Internet]. 2019 Mar 7 [cited 2023 Feb 14];51(3):467–73. Available from: https://pubmed.ncbi.nlm.nih.gov/30604229/10.1007/s11255-018-2051-2 30604229

[20] Pan Z, Zhang L, Niu S, Jiang Y, Li Y. Effect of Febuxostat on Incidence of Contrast-induced Nephropathy in Patients with Coronary Heart Disease Complicated with Hyperuricemia. Chinese Gen Pract [Internet]. 2019 Oct 15 [cited 2023 Feb 14];22(29):3565. Available from: https://www.chinagp.net/EN/10.12114/j.issn.1007-9572.2019.00.172

[21] Mehran R, Aymong ED, Nikolsky E, Lasic Z, Iakovou I, Fahy M et al. A simple risk score for prediction of contrast-induced nephropathy after percutaneous coronary intervention: development and initial validation. J Am Coll Cardiol [Internet]. 2004 Oct 6 [cited 2023 Feb 14];44(7):1393–9. Available from: https://pubmed.ncbi.nlm.nih.gov/15464318/10.1016/j.jacc.2004.06.068 15464318

[22] Iranirad L, Sadeghi MS, Bagheri A, Doostali K, Norouzi S, Hejazi SF et al. Allopurinol prophylactic therapy and the prevention of contrast-induced nephropathy in high-risk patients undergoing coronary angiography: A prospective randomized controlled trial. ARYA Atheroscler [Internet]. 2017 [cited 2023 Feb 11];13(5):230. Available from: /pmc/articles/PMC5774795/PMC5774795 29371869

[23] Tsukamoto S, Okami N, Yamada T, Azushima K, Yamaji T, Kinguchi S et al. Prevention of kidney function decline using uric acid-lowering therapy in chronic kidney disease patients: a systematic review and network meta-analysis. Clin Rheumatol [Internet]. 2022 Mar 1 [cited 2023 Feb 14];41(3):911–9. Available from: https://pubmed.ncbi.nlm.nih.gov/34642880/10.1007/s10067-021-05956-5 34642880

[24] Ma G, Li M, Teng W, He Z, Zhai X, Xia Z. Febuxostat combined with hydration for the prevention of contrast-induced nephropathy in hyperuricemia patients undergoing percutaneous coronary intervention: A CONSORT-compliant randomized controlled trial. Medicine (Baltimore) [Internet]. 2022 Jan 1 [cited 2023 Feb 14];101(4):E28683. Available from: /pmc/articles/PMC8797528/ 10.1097/MD.0000000000028683 PMC8797528 35089218

[25] Pavasini R, Tebaldi M, Bugani G, Tonet E, Campana R, Cimaglia P et al. Contrast Associated Acute Kidney Injury and Mortality in Older Adults with Acute Coronary Syndrome: A Pooled Analysis of the FRASER and HULK Studies. J Clin Med [Internet]. 2021 May 2 [cited 2023 Feb 14];10(10). Available from: https://pubmed.ncbi.nlm.nih.gov/34065642/10.3390/jcm10102151 PMC8156026 34065642

[26] Kamel B, Graham GG, Stocker SL, Liu Z, Williams KM, Carland JE et al. A pharmacokinetic-pharmacodynamic study of a single dose of febuxostat in healthy subjects. Br J Clin Pharmacol [Internet]. 2020 Dec 1 [cited 2023 Feb 14];86(12):2486–96. Available from: https://pubmed.ncbi.nlm.nih.gov/32386239/10.1111/bcp.14357 PMC7688545 32386239

[27] Call J, Sacrinty M, Applegate R, Little W, Santos R, Baki T, et al. Automated contrast injection in contemporary practice during cardiac catheterization and PCI: effects on contrast-induced nephropathy. J Invasive Cardiol. 2006 Oct;18(10):469–74. 17042103

[28] Wang Y, Liu K, Xie X, Song B. Contrast-associated acute kidney injury: An update of risk factors, risk factor scores, and preventive measures. Clin Imaging [Internet]. 2021 Jan 1 [cited 2023 Feb 14];69:354–62. Available from: https://pubmed.ncbi.nlm.nih.gov/33069061/10.1016/j.clinimag.2020.10.009 33069061

[29] Latcha S, Plodkowski AJ, Zheng J, Jaimes EA. Rate and risk factors for AKI after CT scans in a cancer cohort. Clin Nephrol [Internet]. 2019 Mar 1 [cited 2023 Feb 14];91(3):147–54. Available from: https://pubmed.ncbi.nlm.nih.gov/30415653/10.5414/CN109591 PMC6727567 30415653

